# Personalized physiological medicine

**DOI:** 10.1186/s13054-017-1907-7

**Published:** 2017-12-28

**Authors:** Can Ince

**Affiliations:** 1 Department of Intensive Care, Erasmus MC, University Medical Center Rotterdam, ‘s-Gravendijkwal 230, 3015 CE Rotterdam, The Netherlands; 2 Department of Translational Physiology, Academic Medical Center, University of Amsterdam, Meibergdreef 9, 1105 AZ Amsterdam, The Netherlands

## Abstract

This paper introduces the concept of personalized physiological medicine that is specifically directed at the needs of the critically ill patient. This differs from the conventional view of personalized medicine, characterized by biomarkers and gene profiling, instead focusing on time-variant changes in the pathophysiology and regulation of various organ systems and their cellular and subcellular constituents. I propose that personalized physiological medicine is composed of four pillars relevant to the critically ill patient. Pillar 1 is defined by the frailty and fitness of the patient and their physiological reserve to cope with the stress of critical illness and therapy. Pillar 2 involves monitoring of the key physiological variables of the different organ systems and their response to disease and therapy. Pillar 3 concerns the evaluation of the success of resuscitation by assessment of the hemodynamic coherence between the systemic and microcirculation and parenchyma of the organ systems. Finally, pillar 4 is defined by the integration of the physiological and clinical data into a time-learning adaptive model of the patient to provide feedback about the function of organ systems and to guide and assess the response to disease and therapy. I discuss each pillar and describe the challenges to research and development that will allow the realization of personalized physiological medicine to be practiced at the bedside for critically ill patients.

## Background

Randomized controlled clinical trials (RCTs) have failed to provide needed direction for the diagnosis and treatment of the critically ill patient. Such trials, based on the idea that evidence for the treatment of individual patients can only be achieved by demonstrating efficacy of one treatment modality over another in large groups of patients, have not been able to demonstrate effective therapies; at best, the trials (e.g., TRIC, SPLIT, SAFE, PROCESS, ARISE, CHEST, PROWESS, SEPSISPAM, etc.) have shown no difference between groups. These results often lead, in practice, to the mistaken conclusion that there is no difference between the interventions. A more correct conclusion could be that such RCTs, by design, are unable to demonstrate differences in heterogeneous intensive care patients because the physiology of individual patients at the bedside indeed shows differences between various interventions. These shortcomings of RCTs have led to the suggestion that such trials should be abandoned to focus on a more personalized approach for identifying the optimal therapy for each individual patient [1]. This concept has been referred to as “personalized medicine” and has been mainly associated with the measurement of pharmacological biomarkers and genetic profiling with the goal of identifying personalized therapy to result in improved survival benefit (e.g., [2]). However, additional requirements and concepts may be needed for personalized medicine if this concept is to be applied to the critically ill patient. The time-variant changes in (patho)physiology in response to the wide range of disorders with complex interactions between failing organ systems being treated with a variety of drugs and organ-supporting devices distinguishes critically ill patients from other categories of patients (e.g., oncology and cardiology). These considerations lead to the idea that a new form of personalized medicine may need to be designed to meet the specific needs of the critically ill patient.

### Personalized physiological medicine

**Fig. 1:**
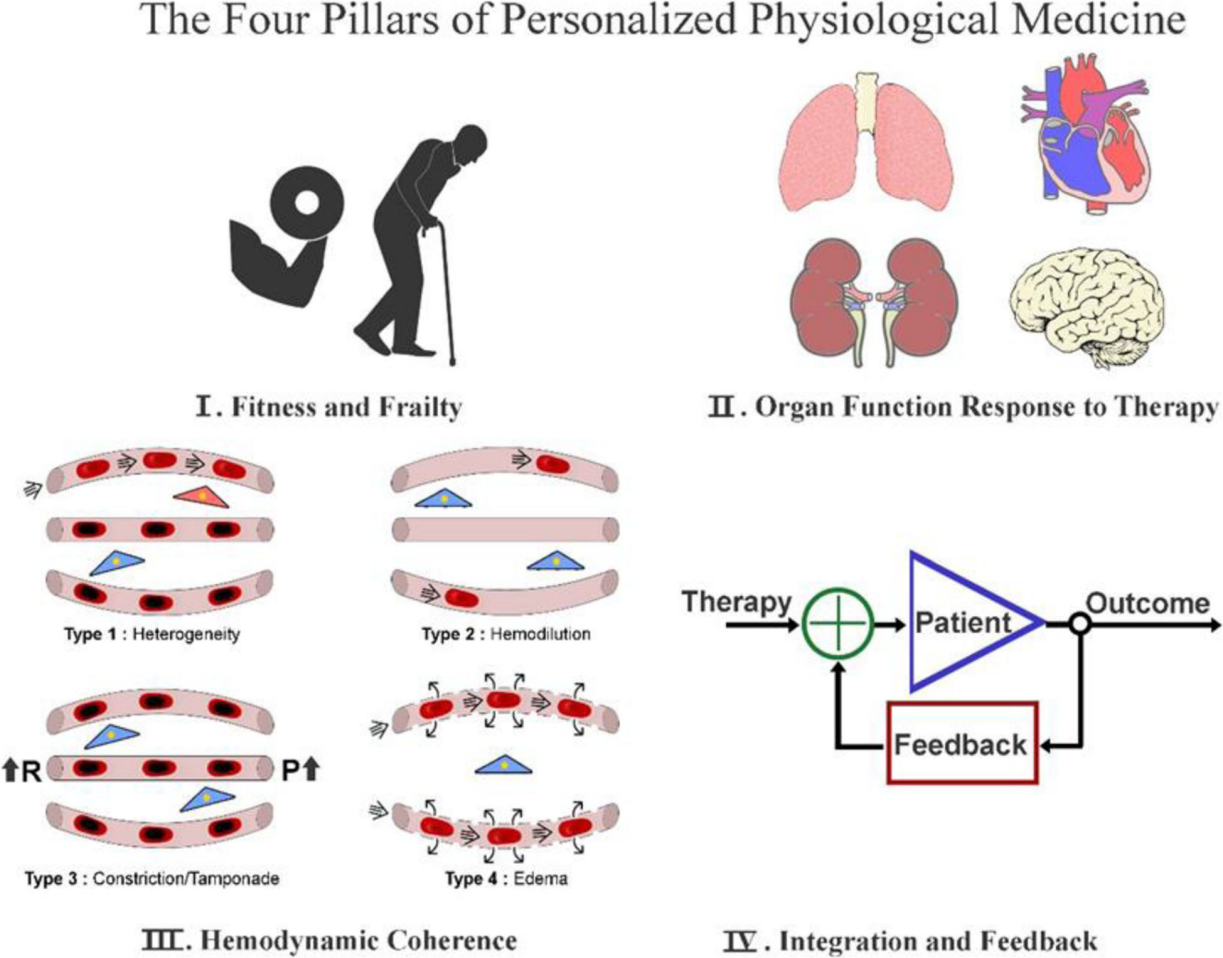
The four pillars of personalized physiological medicine. Pillar I is measurement of the fitness and frailty of the patient and their physiological reserve and fitness to deal with the physiological stress of critical illness. Pillar II concerns measurement of the function of organ systems and their response to therapy as well as their functional capacity and reserve including the immunological, humoral and coagulatory systems. Pillar III concerns the measurement of the hemodynamic coherence between the macro- and microcirculation and parenchymal cells in response to resuscitation. The loss of hemodynamic coherence can be identified by observation of the microcirculation, where type 1 concerns inflammation and infection-induced heterogeneous obstructions of microcirculatory flow, type 2 concerns hemodilution-induced loss of red blood cell filled capillaries, type 3 concerns microcirculatory stasis induced by excessive vasopressor load or raised venous pressures, and type 4 concerns tissue edema (*red* cells are well oxygenated and *blue* cells are hypoxic cells; taken from [35] with permission). Pillar IV is the integration and feedback of the various elements of the personalized physiological medicine modules to provide input in an integrative and time variant holistic manner to identify and assess the success of therapy and severity of organ and cellular dysfunction, as well as identifying the essential parameters in need of correction

Conventional personalized medicine based on genetic profiling and pharmacological biomarkers will need development if they are to be applied to the practical needs of the critically ill patient. The main challenges of this form of personalized medicine will be to obtain genetic and biomarkers in a semicontinuous manner and to link this information to specific organ function allowing targeted therapy to be realized. The genetic profile and transcription factors of the critically ill patient continuously change over time [3]. Levels of pharmacological biomarkers also change continuously over time [4], and the currently available biomarkers of sepsis have been found to lack specificity and sensitivity [5]. These aspects of conventional personalized medicine have prompted the idea that considering physiological variables as biomarkers may provide an essential addition to the needs of the critically ill patient because they relate more closely to the aims of intensive care medicine in terms of providing physiological recovery and organ support [6, 7]. Such a physiological approach to personalized medicine must be focused on the phenotype of the patient as well as on the functional properties of their organs and ultimately their cells as they change over time in response to disease and therapy. From this perspective, I propose here the concept of personalized physiological medicine as being more appropriate in achieving these aims. In doing so, I identify four pillars of personalized physiological medicine on which this concept is based (Fig. 1): 1) fitness and frailty; 2) organ function and response to therapy; 3) hemodynamic coherence; and 4) integration and feedback.

## Pillar I: fitness and frailty

The first pillar of personalized physiological medicine is the assessment of fitness and frailty of the patient to determine their physiological reserve. Although obviously not applicable to critically ill patients, the gold standard for determining fitness is cardiopulmonary exercise testing (CPT), in which cardiovascular stress is imposed by incremental amounts of work and maximum oxygen consumption. Consequently the aerobic threshold is considered as the best index of cardiorespiratory fitness [8] but this has not been applied to critically ill patients. Inadequate exercise has been shown to be a risk factor for sepsis mortality, particularly in diabetics [9]. Exercising critically patients using a bedside cycle ergometer has been shown in survivors to result in improved 6-min walking distance, isometric quadriceps force, and the subjective feeling of well-being following discharge [10]. In experimental studies in septic rats, exercise protected organs from damage and lowered inflammatory mediators [11]. Increasingly, muscle is being recognized as a key hormone secreting organ where myokines, hormones secreted by the exercising muscle, are being shown to play a central role in resolving a host of disease states including cancer and diabetes [12]. Indeed, Montgomery and colleagues demonstrated that muscle wasting during critical illness is directly related to organ failure [13]. That is why developing objective measures of fitness in bed-ridden patients and maintenance of muscle mass by developing exercise modalities during critical illness must be recognized as an important aim in this pillar of personalized physiological medicine.

Extended lack of fitness can translate into frailty, a condition in which homeostatic mechanisms begin to fail, resulting in reductions in the physiological reserve of the neural, renal, skeletal, respiratory, cardiovascular, endocrine, immune, and coagulation systems when challenged by stress [14], such as in critical illness. Several studies have identified phenotypes associated with frailty, including measures related to physical activity, energy, nutritional status, strength, and cognition [15, 16]. The evaluation of frailty as a phenotype in the critically ill patient has been shown in several studies to be of special relevance in the prediction of intensive care unit (ICU) survival (e.g., [17]); frail survivors of critical illness have been shown to experience greater impairment in health-related quality of life and disability compared with those who are not frail [18]. Frailty, defined as a physiologic loss of reserve capacity and resistance to stressors [16, 19], has been quantified in several studies [15]. It is clear that continuous measures of fitness, frailty, and physiological reserve, along with coexisting comorbidity and primary disease, are key input variables defining the phenotype of the patient and therefore represent the first pillar of personalized physiological medicine.

## Pillar II: organ function

The second pillar of personalized physiological medicine involves the function of the organ systems and their response to therapy. Evaluation of the regulatory capacity of the organ systems to stress factors is central because a loss of this regulatory capacity occurs in advance of physical injury to the parenchymal cells associated with upregulation of conventional pharmacological biomarkers. Loss of regulatory capacity represents a window of opportunity for treatment prior to the occurrence of irreversible injury requiring long-term regeneration [6]. Here, providing a physiological challenge to the patient and measuring organ response at the bedside is a central concept in evaluating physiological reserve. For instance, the dobutamine challenge test to evaluate the regulatory capacity of the β-adrenergic system in septic patients was introduced by Vallet and co-workers, who were able to predict survival in septic patients by measuring the response to oxygen delivery, consumption, and extraction [20]. A nonpharmacological version of the dobutamine stress test was explored by Kimmoun et al. to assess the efficiency of cardiac adaptation to septic shock by measuring cardiac contractility reserve-related parameters, including cardiac index, double product, and cardiac power index during the resuscitation procedure [21]. In this context, the heart rate response is also a promising methodology to assess the ability of the autonomic nervous system to regulate cardiovascular responses and assess interorgan communication [22, 23]. The future challenge will be how to therapeutically treat the regulatory capacity of the β-adrenergic system to improve outcome.

Achieving optimal ventilation recruitment and avoiding ventilator-induced lung injury are arguably the main targets in achieving good ventilation-perfusion matching and gas exchange during mechanical ventilation. Adjustment of ventilator settings, assessing deleterious effects of often-used therapy, including fluid therapy and mechanical ventilation, and evaluating the direct effects of therapies directed at the lung itself, such as nebulization of antibiotics [24], anticoagulants [25], anti-inflammatory [26] and vasoactive compounds [27], truly requires a personalized approach in which bedside lung function evaluation is essential. Although several lung function parameters are available at the bedside (e.g., airway resistance, tidal volume, end-expiratory lung volume, intrinsic positive end-expiratory pressure (PEEP), compliance, dead space, and volumetric capnography) and novel clinical methodologies such as electric impedance tomography are being developed [28], essential lung function parameters directly related to the capacity of the lung to achieve gas exchange are lacking. A need exists for the quantitative assessment of functional residual capacity (FRC), inhomogeneity of ventilation, and ventilation-perfusion matching. Indeed the importance of such measurements have been demonstrated in experimental models of acute lung injury (ALI) where the effects of respiratory movements could be directly observed in exposed mice lungs using dark-field intravital microscopy [29]. Measurement of these parameters has classically required the quantitative measurement of the washout of inert indicator gases requiring the use of complex mass spectrometry [30] at the bedside [31]. More practical measurement of these parameters at the bedside is currently under investigation (e.g., [32]). In addition to these volumetric measures, more comprehensive physical properties of the lung tissue itself are required beyond conventional dynamic compliance and airway resistance measures. Such information can be obtained, for example, by the forced oscillation technique in which the frequency-dependent impedance of the complete pulmonary system can be obtained, providing detailed information about the mechanical properties of the lung (e.g., [33]). It is clear from these considerations that there is a need to further develop techniques to measure these pulmonary parameters and to integrate them into a single monitoring platform to meet the requirements of this pillar of personalized physiological medicine.

Measuring kidney function is a specific challenge in intensive care management. This has typically been limited to the measurement of urine production and creatinine levels, both of which are considered inadequate indicators of kidney function. As a result, there has been a surge in renal pharmacological biomarker research. Although these biomarkers have been effective in identifying renal injury, they have not yet proven successful in guiding therapy. Their time-variant changes and their sensitivity only to advanced renal injury led us to develop the concept of physiological biomarkers of acute kidney injury (AKI) [6]. We proposed that such physiological biomarkers be related to renal hemodynamics and regulation, microcirculation and oxygenation, and tubular function because these are expected to be altered in advance of an injury, thereby identifying a window of therapeutic efficacy. Consistent with these concepts, Ronco and co-workers developed methodologies to measure renal physiological reserve, which was defined as the capacity of the kidney to increase the rate of glomerular filtration in response to a physiological stress; they proposed the administration of a fixed protein load for this purpose [34]. Using a similar concept to measure the functional capacity of the kidney, Chawla and co-workers administered furosemide to stimulate urine production and found that the furosemide stress test was much more sensitive in predicting stage 3 AKI than pharmacological biomarkers [35]. From these examples it is clear that there is a concerted effort to establish a functional platform to more comprehensively monitor organ function and assess the capacity to regulate functional reserve in real time as an essential goal for personalized physiological medicine. Recent advanced in ultrasound such as contrast-enhanced ultrasound may make such sensitive monitoring of the renal microcirculation feasible in patients [36].

## Pillar III: hemodynamic coherence

Resuscitation aims at normalizing systemic hemodynamic variables, such as stroke volume or blood pressure, with the expectation that a parallel improvement will occur in the perfusion and oxygenation of the microcirculation feeding the tissue beds of the organ systems. Homeostatic coupling between the systemic circulation and the microcirculation is essential for such an expectation to be met; in addition to the primary disease, resuscitation fluids and medications themselves can adversely affect this regulation. We have termed the required coupling between the macro- and microcirculation essential for successful resuscitation based on the correction of systemic hemodynamic variables as “hemodynamic coherence” [37]. Loss of hemodynamic coherence can occur if the factors affecting the microcirculation are not corrected by the resuscitation procedure focused on correction of the macrocirculation by resuscitation following shock. Such factors affecting the microcirculation can include immunological and/or factors affecting endothelial, leucocyte, and red blood cell function. In this context, its manifestation should be regarded as a dynamic process depending on the interactions between disease, therapy, and time. Whether the correction of systemic hemodynamic variables achieves adequate microcirculatory and tissue perfusion is often unknown and may manifest at the bedside as the patient being unresponsive. Such a situation prompts the clinician to administer even more fluids and medications, potentially causing harm. Assessment of the presence or absence of hemodynamic coherence requires the simultaneous measurement of the response of the macro- and the microcirculation. The microcirculation can be effectively visualized in the sublingual area using hand-held vital microscopy (e.g., [38]), which allows the parallel improvement in the microcirculation to resuscitation efforts based on the response of systemic parameters to be verified [39]. Identification of the presence or absence of hemodynamic coherence and the response of the microcirculation to therapy forms the third pillar of personalized physiological medicine because it assesses the physiological coupling between the various compartments in the hierarchy of the circulation to achieve uniform resuscitation. Loss of hemodynamic coherence at the level of the microcirculation can be divided into four types (Fig. 1). Type 1 loss of hemodynamic coherence is characteristic of states of sepsis, in which inflammatory mediators and oxidative and nitrosative stress factors cause endothelial and erythrocyte injury resulting in obstruction of the capillaries. This causes a heterogeneous microcirculatory flow and functional shunting in parts of the microcirculation, resulting in reduced oxygen extraction capacity characteristic of sepsis [40]. Type 2 loss of hemodynamic coherence occurs when an excessive volume of fluids is given in an attempt to correct systemic variables, such as stroke volume and blood pressure. While systemic variables may be normalized, hemodilution causes reduced viscosity and dilution of the blood, both of which cause a reduction in capillary filling. This increases the diffusion distances between oxygen-carrying erythrocytes and tissue cells, thereby reducing the oxygen delivery capacity of the microcirculation and its oxygen extraction capacity [39]. Type 3 loss of hemodynamic coherence is the condition where high levels of vasopressors intended to improve blood pressures can paradoxically cause constriction of microcirculatory blood flow [41]. Similarly, microcirculatory impediment of flow can occur when high venous pressures are targeted, resulting in microcirculatory flow restriction due to tamponade [42]. Type 4 loss of hemodynamic coherence occurs as a result of edema (e.g., in burns [43] and in malaria [44]) in which leaky vessels also cause increased diffusion distances and a reduction in oxygen extraction.

## Pillar IV: feedback and integration

Personalized physiological medicine directly relates to the practice of intensive care in supporting organ function and restoring homeostasis. Concepts from systems and control engineering, in which integration and feedback are central for the control of complex systems [22], are important to consider. To this end, I define the fourth pillar of personalized physiological medicine as the integration of the modules in the aforementioned three pillars to provide feedback on the functional activity of different physiological compartments, to identify the functional state and stability of the system, and to provide practical feedback to guide therapy and ensure resolution of the unstable patient prior to the development of irreversible states of critical illness.

Intensivists are confronted with an overwhelming amount of patient data, including historical clinical information as well as continuous online data regarding the condition of the patient, which changes from moment to moment [45]. Decisions based on assessment of these data are based on clinical experience as well as evidence from trials and knowledge of the literature; however, this assessment relies more on the subjective judgment of the physician rather on a strict analysis of data. Various initiatives have been formulated to integrate and simplify the vast amount of data being generated from the patient, describing both the condition of the patient (e.g., APACHE, SAPS and SOFA) and that of specific organ systems (e.g., AKIN and KDIGO for AKI). Currently, more sophisticated predictive methodologies are being developed, making use of complex mathematics such as chaos and complexity theory [46]. Almost without exception, these methods are used to evaluate the severity of disease without providing any insight into the physiologic basis for the condition of the patient. These approaches neither identify a given physiological parameter in need of correction nor identify optimal therapy and provide feedback for a therapeutic maneuver in a goal-directed manner.

For these reasons, the fourth pillar of personalized physiological medicine requires not only a predictive environment to describe the condition of the patient, but a more comprehensive mathematical model directly related to the function of the organ systems from a systems engineering and integrative systems physiological perspective. Here, measuring the interactions between the various physiological compartments, including the immune and humoral systems as well as the cellular and ultimately even the genetic profile, stemming from the previous three pillars of personalized physiological medicine should provide a holistic description of the physiological state of the patient and, more importantly, provide practical feedback for identifying the need, response, and success of therapy. Such an approach requires adaptive modeling in which the model is continuously responding, considering time-variant changes and providing an optimal model for patient care (e.g., [47]). Central to the model should be the measures of organ function of pillar 2 to provide the needed feedback to evaluate organ and therapy support and interactions between the different physiological compartments. In this way, the model should be capable of assessing the stability of the system so that successful weaning from an assisted mode can be accomplished, in which therapy and organ-supporting devices are successful in achieving eventual independent organ function. It can even be conceived that such models can become closed loop control systems for control of specific parts of the support system (e.g., [48]).

## Conclusion

Personalized medicine is a developing trend for the future of intensive care medicine. However, the practical implementation of this concept, if limited to the use of genetic screening and pharmacological biomarkers, however appealing, is still in need of considerable development. I therefore propose a personalized physiological approach which I argue is much more suited to the requirements of critically ill patients. I have presented four pillars of personalized physiological medicine to address the full spectrum of this idea. This classification allows a modular approach, as its various aspects are under development in sometimes unrelated areas of critical care medicine. Integration of the concepts will provide a true challenge for the future, requiring collaboration between clinicians, physiologists, and engineers; the realization of bedside instruments to practice personalized physiological medicine remains a real challenge to industry. Nevertheless, I anticipate that the road map outlined in this paper may provide a conceptual framework within which critically ill patients will benefit from the promises of personalized medicine.
